# A structured understanding of cellobiohydrolase I binding to poplar lignin fractions after dilute acid pretreatment

**DOI:** 10.1186/s13068-018-1087-y

**Published:** 2018-04-04

**Authors:** Lan Yao, Chang Geun Yoo, Xianzhi Meng, Mi Li, Yunqiao Pu, Arthur J. Ragauskas, Haitao Yang

**Affiliations:** 1 School of Pulp & Paper Engineering, Hubei University of Technology, Wuhan, 430068 China; 2 Hubei Provincial Key Laboratory of Green Materials for Light Industry, Hubei University of Technology, Wuhan, 430068 China; 3 Joint Institute for Biological Sciences, Biosciences Division, Oak Ridge National Laboratory, Oak Ridge, TN 37831 USA; 4 Department of Chemical and Biomolecular Engineering, The University of Tennessee, Knoxville, TN 37996-2200 USA; 5 Department of Forestry, Wildlife and Fisheries, Center for Renewable Carbon, Institute of Agriculture, The University of Tennessee, Knoxville, TN 37996-2200 USA

**Keywords:** Cellobiohydrolase I, Enzyme binding, Lignin, Dilute acid pretreatment, Poplar

## Abstract

**Background:**

Cellulase adsorption to lignin is considered a cost barrier for bioethanol production; however, its detailed association mechanism is still not fully understood. In this study, two natural poplar variants with high and low sugar release performance were selected as the low and high recalcitrant raw materials (named *L* and *H*, respectively). Three different lignin fractions were extracted using ethanol, followed by *p*-dioxane and then cellulase treatment from the dilute acid pretreated poplar solids (fraction 1, 2, and 3, respectively).

**Results:**

Each lignin fraction had different physicochemical properties. Ethanol-extracted lignin had the lowest weight average molecular weight, while the molecular weights for the other two lignin fractions were similar. ^31^P NMR analysis revealed that lignin fraction with higher molecular weight contained more aliphatic hydroxyl groups and less phenolic hydroxyl groups. Semi-quantitative analysis by 2D HSQC NMR indicated that the lignin fractions isolated from the natural variants had different contents of syringyl (S), guaiacyl (G) and interunit linkages. Lignin extracted by ethanol contained the largest amount of S units, the smallest amounts of G and *p*-hydroxybenzoate (PB) subunits, while the contents of these lignin subunits in the other two lignin fractions were similar. The lignin fraction obtained after cellulase treatment was primarily comprised of β-*O*-4 linkages with small amounts of β-5 and β–β linkages. The binding strength of these three lignin fractions obtained by Langmuir equations were in the order of *L*_1_ > *L*_3_ > *L*_2_ for the low recalcitrance poplar and *H*_1_ > *H*_2_ > *H*_3_ for the high recalcitrance poplar.

**Conclusions:**

Overall, adsorption ability of lignin was correlated with the sugar release of poplar. Structural features of lignin were associated with its binding to CBH. For natural poplar variants, lignin fractions with lower molecular weight and polydispersity index (PDI) exhibited more CBH adsorption ability. Lignins with more phenolic hydroxyl groups had higher CBH binding strength. It was also found that lignin fractions with more condensed aromatics adsorbed more CBH likely attributed to stronger hydrophobic interactions.

**Electronic supplementary material:**

The online version of this article (10.1186/s13068-018-1087-y) contains supplementary material, which is available to authorized users.

## Background

Lignin is a major component in the cell wall of terrestrial plants and usually constitutes about 15–30% of its total dry weight. It plays an important role in the structural integrity and protection from microorganism attack [1]. Unlike plant polysaccharides (i.e., cellulose and hemicellulose), lignin is a three-dimensional cross-linked macromolecular polymer composed of phenylpropanoid units, typically derived from guaiacyl, syringyl and/or *p*-hydroxyphenyl, connected by C–C and C–O interunit linkages [2]. The contents of guaiacyl (G), syringyl (S) and *p*-hydroxyphenyl (H) units in lignin varies from species to species, which are commonly used to categorize the three major types of biomass, softwood lignin mainly contains G unit, hardwood lignin is composed of both G and S units, and the lignin from herbaceous origins is composed of G and S units as well as lesser amounts of H units [3].

Lignin is considered as a major barrier that hinders the commercialization process of biomass to biofuel production, as it is chemically and physically associated with cellulose and hemicellulose [4]. The negative effects of lignin on cellulase performance were first identified in 1980s [5, 6]. Recently, increased research efforts have been conducted in this field to make biomass utilization economically and technically feasible [7]. These studies have reported several important findings including the fact that a higher phenolic hydroxyl content can result in an increased lignin cellulase adsorption capacity [8–10], and a higher carboxylic acid group content of the associated lignin can enhance the enzymatic hydrolysis of lignocellulosic biomass [11, 12]. Overall, it has been proposed that cellulase binds to lignin through three major interactions: hydrophobicity [11, 13], hydrogen bonding [14] and electrostatic interactions [12]. Furthermore, it was reported that lignin composition could also influence the enzymatic hydrolysis process [3]. However, inconsistent results were reported about the effect of S/G ratio on cellulase adsorption. Some researchers found that a high S/G ratio is favorable for the hydrolysis yield, because of the higher binding capability of G over S to cellulase [15, 16], while others indicated that lignin with higher S/G ratio had higher cellulase binding ability [17]. As a result, further studies are needed to clarify the mechanisms leading to the inconsistent results.

The aerobic fungus *Trichoderma* is a common source of enzymes used in cellulase production. The research on cellulase from *Trichoderma reesei* and its binding to lignin have been studied in recent years. Nonaka found that lignin from steam-exploded pretreated eucalyptus adsorbed more *T. reesei* cellulase than lignin from native eucalyptus [18]. Adsorption of *T. reesei* cellulase on softwood lignin-based lignophenol indicated that cellulase adsorption on lignin is single-layered and phenolic hydroxyl could enhance cellulase binding [19]. Binding of CBH I and EG II purified from *T*. *reesei* to steam pretreated softwood (SPS) were compared. The results showed that more of CBH I was absorbed by SPS [20]. However, reports on cellulase from *Trichoderma longibrachiatum* are limited.

In this study, two 4-year-old natural poplar variants harvested under uniform conditions from Clatskanie, Oregon were selected as the raw materials. The two native poplar variants showed different glucose release performance upon treatment with cellulase. During enzymatic hydrolysis, the low recalcitrance poplar (*L*) released twice the amount of glucose than the high recalcitrance poplar (*H*) did [21]. Up till now, detailed mechanism about cellulase binding to lignin still has not been fully elucidated, which is largely attributable to the structural complexity and heterogeneity of lignin. Hence, a simple fractionation approach of lignin is needed. Here, solvents were chosen based on their chemical nature, polarity and ability to form hydrogen bonds with lignin. To investigate the effects of lignin characteristics on cellulase–lignin interactions, the acid pretreated poplar was sequentially extracted to generate lignin fraction samples with different reactivity or structural properties. Pretreated poplar samples were extracted with ethanol to obtain the first lignin fraction. Then, the solid residue was further extracted with 96% dioxane to acquire the second lignin fraction and the third fraction of lignin was collected by treating the remaining solid residue with cellulase followed by extraction with dioxane. Fourier transform infrared (FT-IR), gel permeation chromatographic (GPC) and nuclear magnetic resonance (NMR) were employed to study the structural characteristics of different lignin samples. Cellobiohydrolase I (CBH), which was the protein in the filtrates of cultured fungi responsible most for cellulose hydrolysis [22], was then used to investigate the binding properties of the three different lignin fractions. Finally, the relationship between lignin structural features and CBH binding properties was analyzed accordingly.

## Results and discussion

### FT-IR analysis

**Fig. 1:**
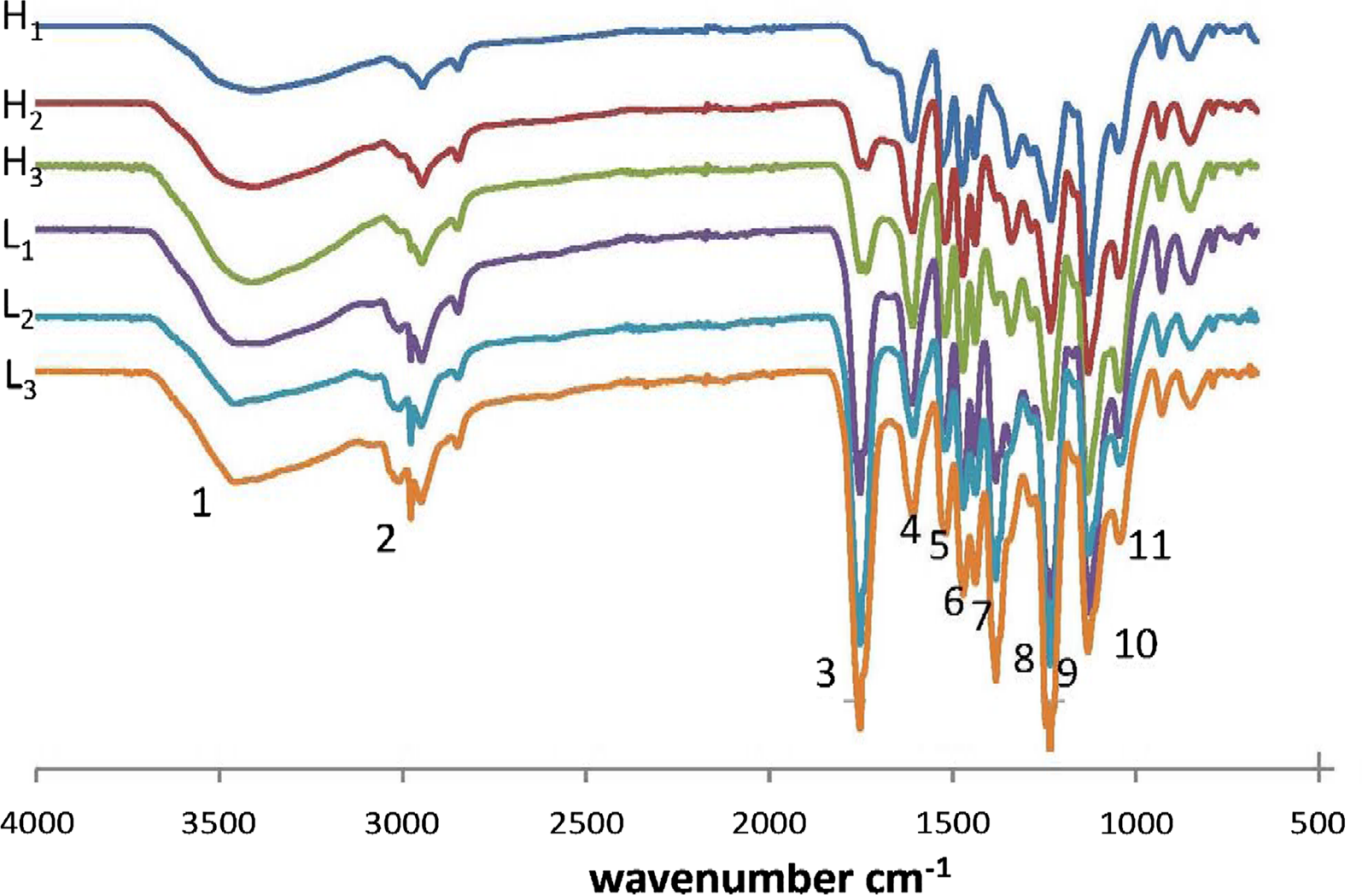
FT-IR spectra of lignin fractions from natural poplar variants

The FT-IR spectra of six lignin fractions are shown in Fig. 1. The assignments of major signals were based on published literatures [15, 23, 24]. The strong signal at 3410 cm^−1^ was ascribed to hydroxyl bond (O–H) stretching and the absorption at 2938 cm^−1^ was from C–H stretching vibrations. Signals centered at about 1596, 1513 and 1424 cm^−1^ corresponding to aromatic rings were clearly observed in all lignin samples.

The absorption at 1459 cm^−1^ was attributed to the C–H asymmetric deformations. The signals at 1324 and 1112 cm^−1^ were corresponded to syringyl/condensed guaiacyl and aromatic C–H deformation of syringyl unit, respectively. The absorption bands at around 1215 cm^−1^ belonged to C–O stretching of guaiacyl unit. It indicated that the lignin samples from natural poplar variants were G–S type. The band at 1030 cm^−1^ was attributed to aromatic C–H in-plane deformation vibrations.

The relative signal intensities of various functional groups were calculated as ratios referenced to the intensity of band at 1513 cm^−1^ (Table 1). It was found that the most obvious difference was the signal at around 1738 cm^−1^, which was ascribe to C=O in unconjugated ketones. This substructure was the least in the ethanol-extractable lignin fraction and the most in the lignin fraction recovered after cellulase treatment in both poplars. Previous studies on fractionation of degraded lignins from the black liquor of *Eucalyptus pellita* also revealed that lignin fraction extracted by *n*-propanol showed stronger absorption at 1711 cm^−1^ [25]. In addition, the singal intensity of C–O was the least in the ethanol-extractable lignin fraction, while those of the other two lignin fractions were similar.

**Table 1:** Signal assignment and relative intensities of lignin fractions from natural poplar variants in FT-IR spectra

No.	Assignment	Wavenumber (cm^−1^)	*H*_1_	*H*_2_	*H*_3_	*L*_1_	*L*_2_	*L*_3_
1	Hydroxyl group	3410	0.49	0.68	0.75	0.57	0.73	0.73
2	C–H stretching	2938	0.47	0.67	0.64	0.65	0.89	0.85
3	C=O in unconjugated ketone	1738	0.13	0.54	0.69	1.29	2.36	2.25
4	Aromatic ring	1596	0.83	0.98	1.00	0.86	0.93	0.95
5	Aromatic ring	1513	1.00	1.00	1.00	1.00	1.00	1.00
6	C–H deformation	1459	1.14	1.27	1.27	1.24	1.37	1.36
7	Aromatic ring	1424	0.95	1.06	1.10	1.10	1.34	1.35
8	Syringyl and condensed guaiacyl	1324	1.01	1.06	1.06	1.05	1.06	1.08
9	C–O stretching	1215	1.39	1.66	1.66	1.85	2.41	2.37
10	Aromatic C–H deformation in syringyl	1112	1.89	1.96	2.00	1.85	1.76	1.78
11	C–O–C stretching	1030	0.91	1.29	1.39	1.01	1.14	1.10

The relative intensity was calculated as the ratio of the intensity of the band to the intensity of band at 1513 cm^−1^

### Molecular weight analysis

Prior to GPC analysis, the lignin fractions were acetylated to facilitate their dissolution in THF [26]. The weight average molecular weights (*M*_*w*_), number average molecular weights (*M*_*n*_), and polydispersity index (PDI) (*M*_*w*_/*M*_*n*_) of the lignins are presented in Table 2. It was found that the ethanol-extractable lignin fractions from the dilute acid pretreated poplar had the lowest molecular weights. The *M*_*w*_ for *L*_1_ and *H*_1_ lignin fractions were 1649 and 1570 g/mol, respectively. The *M*_*w*_ of the other two lignin fractions were higher (4437, 3354, 4196 and 3975 for *L*_2_, *L*_3_ and *H*_2_, *H*_3_ lignin fractions, respectively) than the ethanol-extractable lignin fractions. These results indicated that the ethanol-extracted lignin fractions could represent the low molecular weight lignin, while the other two fractions were representative of the bulk and residual lignin. The PDI of all lignin samples was narrow (less than 2) and significant difference between the variants was not observed. The results were consistent with previous studies on lignin from poplar [27], which indicated that after dilute acid pretreatment, the weight average molecular weight was around 7500, while number average molecular weight was about 3000. Due to the fractionation of lignin from poplar, lignin fractions in the present study showed narrower PDI and lower molecular weight. The lower molecular weight in the present study was also caused by the lower solid/liquid ratio and more acid used in the pretreatment process.

**Table 2:** Molecular weights and PDI of lignin fractions from dilute acid pretreated natural poplar variant

Sample	*M*_*n*_	*M*_*w*_	PDI (*M*_*w*_/*M*_*n*_)
*L*_1_	1007 ± 35	1649 ± 113	1.64 ± 0.06
*L*_2_	2310 ± 3	4437 ± 1	1.92 ± 0.00
*L*_3_	1828 ± 97	3354 ± 248	1.83 ± 0.04
*H*_1_	886 ± 2	1570 ± 12	1.77 ± 0.00
*H*_2_	2263 ± 8	4196 ± 37	1.86 ± 0.01
*H*_3_	2336 ± 131	3975 ± 277	1.78 ± 0.02

### ^31^P NMR analysis

**Fig. 2:**
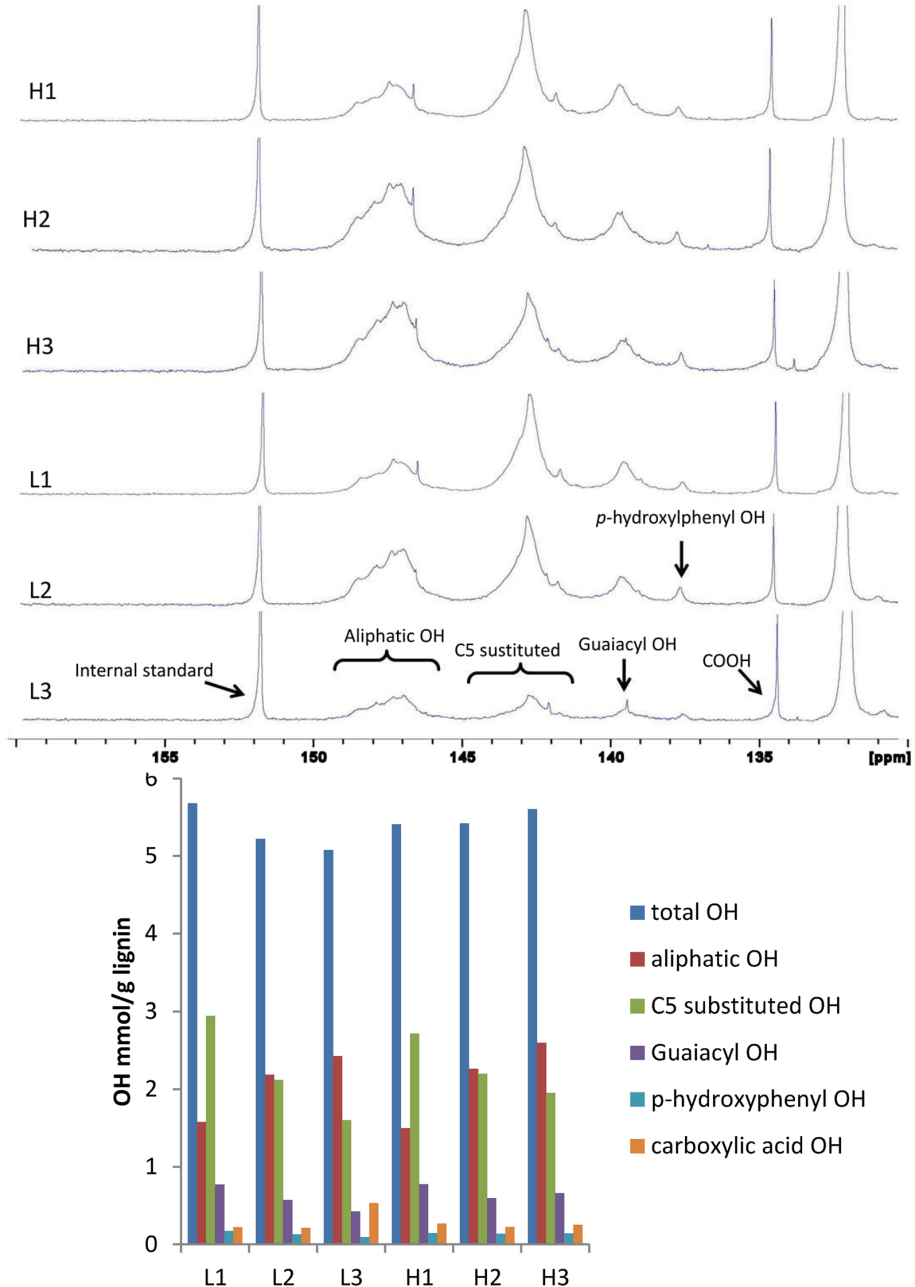
^31^P NMR spectra and analysis results of lignin fractions from natural poplar variants

In this study, to investigate the major hydroxyl group contents in each lignin fraction, the lignin samples were derivatized with 2-chloro-4,4,5,5-tetramethyl-1,3,2-dioxaphospholane (TMDP) [25]. The phosphitylated hydroxyl groups were measured by integrating the area of individual peaks compared to the known concentration of the internal standard *N*-hydroxy-5-norbornene-2,3-dicarboximide [28]. Figure 2 presents the contents of aliphatic hydroxyl groups, C_5_ substituted guaiacyl/syringyl phenolics and guaiacyl phenolic groups, *p*-hydroxyphenyl groups and carboxylic acids hydroxyl groups.

The results indicated that the aliphatic and C_5_ substituted phenolic (mainly from syringyl aromatics) hydroxyls were the major hydroxyls of all the lignin fractions. The amount of aliphatic hydroxyls increased in the order of *L*_1_ < *L*_2_ < *L*_3_, *H*_1_ < *H*_2_ < *H*_3_. On the other hand, the content of C_5_ substituted phenolics and syringyl hydroxyls increased in the opposite manner: *L*_3_ < *L*_2_ < *L*_1_, *H*_3_ < *H*_2_ < *H*_1_. Lignin fractions from acid pretreated poplar with higher molecular weights contained more aliphatic OH and less C5 substituted phenolics as previously reported [29]. It has been reported that the formation of lignin with relatively small molecular weight involves the formation of new phenolic hydroxyl groups and the elimination of aliphatic hydroxyl groups [30]. The ethanol-extracted lignin fractions had more C_5_ substituted phenolic groups (mainly from syringyl aromatics) than aliphatic hydroxyl groups. The dioxane-extracted lignin fractions exhibited comparable phenolic/hydroxyl characteristics, while the amount of aliphatic hydroxyl groups was higher than that of C_5_ substituted phenolic groups (mainly from syringyl aromatics) in the third lignin fractions. The hydroxyl group contents in the same lignin fractions from different natural poplar variants did not show significant difference, except the content of carboxylic acid hydroxyls in *L*_3_.

### HSQC NMR analysis

To explore the structural characteristic of lignin fractions from natural poplar variants, 2D HSQC NMR analysis was conducted (Additional file 1: Figure S1). The cross peaks were assigned according to literatures [23, 27, 31, 32].

The signals from the aromatic region (*δ*_C_/*δ*_H_ 160–90/8.0–5.5 ppm) were assigned mainly to the unsubstituted carbons in aromatic rings of lignin units. The syringyl unit showed correlations for C_2,6_/H_2,6_ centered at around *δ*_C_/*δ*_H_ 103.1/6.60 ppm. The C_2,6_/H_2,6_ correlation of the α-oxidized syringyl unit was shifted to *δ*_C_/*δ*_H_ 106.0/7.23 ppm. Cross peaks of condensed syringyl unit were found at around *δ*_C_/*δ*_H_ 105.4/6.42 ppm [8]. The guaiacyl unit was evidenced by cross peaks for C_2_/H_2_, C_5_/H_5_ and C_6_/H_6_ centered at *δ*_C_/*δ*_H_ 110.2/6.91, 114.7/6.69, and 118.4/6.75 ppm, respectively. Cross peaks of condensed guaiacyl unit were found at around *δ*_C_/*δ*_H_ 112.0/6.65 ppm [8]. Signal for *p*-hydroxyphenyl benzoate unit was observed by C_2,6_/H_2,6_ correlation at *δ*_C_/*δ*_H_ 130.9/7.63 ppm. The quantitative information of various substructures in lignin fractions from natural poplar variants are presented in Table 3. The content of total syringyl unit was the most in the ethanol-extracted lignin fractions, and the contents in the other two lignin fractions decreased slightly. On the contrary, the contents of G unit were higher in NO.2 and NO.3 lignin fractions (16–19%) than that of NO.1 lignin (11–15%). As a result, the S/G ratios of ethanol-extracted lignin fractions were higher than that of the other two lignin fractions. The *p*-hydroxybenzoate, which may take part in lignification of cell wall [33], was also observed in all lignin samples with its contents higher in dioxane-extracted and cellulase-treated lignin fractions.

**Table 3:** Semi-quantitative information of lignin samples

Lignin substructure	*δ*_C_/*δ*_H_ (ppm)	*L*_1_	*L*_2_	*L*_3_	*H*_1_	*H*_2_	*H*_3_
		%^a^	%^a^	%^a^	%^a^	%^a^	%^a^
S	103.1/6.60	32.4 ± 1.8	49.1 ± 0.3	48.2 ± 0.8	41.1 ± 0.7	38.5 ± 0.2	34.8 ± 0.3
S’	106.0/7.23	7.1 ± 0.1	7.7 ± 0.0	7.1 ± 0.6	8.5 ± 0.2	5.7 ± 0.4	5.5 ± 0.0
S condensed	105.4/6.42	45.3 ± 1.2	26.8 ± 0.0	29.0 ± 0.0	38.5 ± 0.5	39.8 ± 0.1	40.7 ± 0.1
Total S	–	84.6 ± 0.3	83.6 ± 0.3	84.3 ± 0.2	88.1 ± 0.0	83.9 ± 0.4	81.0 ± 0.1
G	110.2/6.91	8.1 ± 0.1	14.5 ± 0.0	13.5 ± 0.4	9.5 ± 0.0	14.5 ± 0.3	16.6 ± 0.0
G condensed	112.0/6.65	7.3 ± 0.6	2.5 ± 0.4	2.1 ± 0.2	2.4 ± 0.0	1.6 ± 0.0	2.4 ± 0.0
Total G	–	15.4 ± 0.4	17.0 ± 0.4	15.7 ± 0.2	11.2 ± 0.0	16.1 ± 0.4	19.0 ± 0.1
PB	130.9/7.63	7.5 ± 0.0	7.9 ± 0.2	9.5 ± 0.3	4.2 ± 0.2	6.0 ± 0.7	6.1 ± 0.4
S/G	–	5.5 ± 0.1	4.9 ± 0.1	5.4 ± 0.1	7.4 ± 0.0	5.2 ± 0.1	4.3 ± 0.0
β-*O*-4	71.6/4.85	66.5 ± 0.0	71.2 ± 0.7	76.2 ± 2.7	59.5 ± 1.0	70.8 ± 2.0	74.9 ± 1.0
β-5	87.0/5.43	11.7 ± 0.0	10.7 ± 0.6	6.7 ± 0.8	17.1 ± 1.5	9.5 ± 0.7	7.8 ± 0.8
β–β	85.0/4.63	21.8 ± 0.0	18.1 ± 1.3	17.2 ± 1.8	23.4 ± 0.4	19.8 ± 0.3	17.3 ± 0.2

^a^ Amount of specific functional group was expressed as percentage of S + G for S, G and PB; of total side chain for β-*O*-4, β-5 and β–β

In the aliphatic region (*δ*_C_/*δ*_H_ 90–45/6.0–2.0 ppm) of NMR spectra, the cross peaks of methoxyl and major interunit linkages such as β-aryl-ether (β-*O*-4), phenylcoumaran (β-5) and resinol (β–β) were the most prominent ones. The C–H correlations in β-*O*-4 substructure were confirmed by C_*α*_/H_*α*_ at *δ*_C_/*δ*_H_ 71.6/4.85 ppm (β-*O*-4 linked to a S unit) and 71.1/4.74 ppm (β-*O*-4 linked to a G unit), C_*β*_/H_*β*_ at *δ*_C_/*δ*_H_ 85.9/4.11 ppm (β-*O*-4 linked to a S unit) and 83.4/4.28 ppm (β-*O*-4 linked to a G unit), C_*γ*_/H_*γ*_ at *δ*_C_/*δ*_H_ 59.7/3.67 ppm. The presence of phenylcoumaran was well-resolved for C_*α*_/H_*α*_ correlations at around *δ*_C_/*δ*_H_ 87.0/5.43 ppm. Lignin resinol was also observed by its C–H correlations at *δ*_C_/*δ*_H_ 85.0/4.63 ppm (C_*α*_/H_*α*_), 53.6/3.06 ppm (C_*β*_/H_*β*_), and 70.9/4.18 ppm (C_*γ*_/H_*γ*_). Apparently, signals associated with β-*O*-4 interunit linkages prominently appeared in all these lignin fractions, and the relative content was increased from *L*_1_/*H*_1_ lignin to *L*_3_/*H*_3_ lignin, while the relative contents of phenylcoumaran and resinol linkages were decreased.

### CBH adsorption to lignins by Langmuir equation

To assess the binding ability between CBH and isolated lignin fractions, Langmuir adsorption isotherms of CBH with the different lignins were determined and are presented in Table 4. Binding strength is a parameter to estimate the enzymes onto lignins by incorporating both maximum adsorption capacity and equilibrium constant. The results showed that the binding strength of lignin fractions were 670, 176, and 417 ml/g lignin for *L*_1_, *L*_2_ and *L*_3_, and 556, 378 and 370 ml/g lignin for *H*_1_, *H*_2_ and *H*_3_, respectively. The highest binding strength was observed for *L*_1_ (670 ml/g) and *H*_1_ (556 ml/g). The mass yields of each lignin fraction were 46.7, 50.0 and 3.3% for low recalcitrance poplar, while those from high recalcitrance poplar were 50.4, 46.8 and 2.8%, respectively. Combining the results of lignin fraction yield and binding strength, it was found that the total binding ability of lignin from the two poplars were 414 ml/g (low recalcitrance poplar) and 467 ml/g (high recalcitrance poplar). The variations in the adsorption parameters for the lignin samples might be due to the different structure characteristic.

**Table 4:** Langmuir adsorption isotherm parameters from CBH adsorption to lignins

	*E*_max_ (mg/g)	*K*_ads_ (ml/mg)	Binding strength (ml/g lignin)	*R* ^2^
*L*_1_	89.29	7.47	670	0.85
*L*_2_	33.44	5.25	176	0.99
*L*_3_	49.75	8.38	417	0.99
*H*_1_	52.91	10.5	556	0.99
*H*_2_	497.51	0.76	378	0.86
*H*_3_	188.68	1.96	370	0.99

### The correlations between lignin structural characteristic and non-productive CBH adsorption factors

**Fig. 3:**
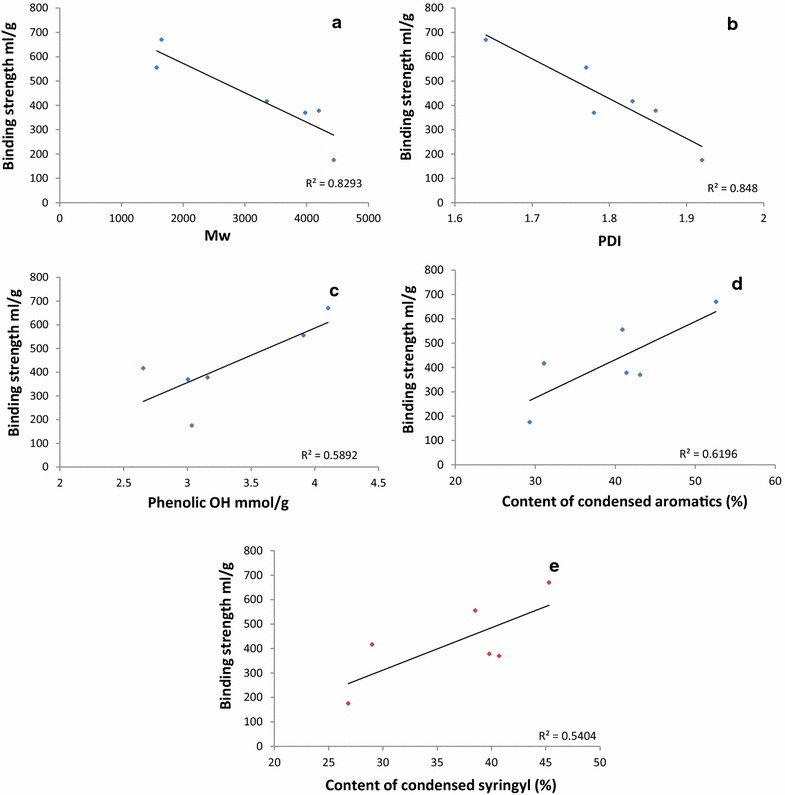
Relationship between lignin physicochemical properties and binding strength between lignin and CBH (**a** relationship between lignin Mw and binding strength of lignin with CBH; **b** relationship between lignin PDI and binding strength of lignin with CBH; **c** relationship between the phenolic hydroxyl group contents and binding strength of lignin with CBH; **d** relationship between contents of condensed aromatics and binding strength of lignin with CBH; **e** relationship between contents of condensed syringyl unit and binding strength of lignin with CBH)

The correlations of molecular weight and PDI of lignin on cellulase have been stated in previous studies [15, 21, 34]; however, the conclusions were not consistent. In the present study, lignin fraction from natural variant poplar with lower molecular weight and PDI showed more CBH binding ability, suggesting that lignin fraction with smaller molecular weight and lower PDI favoring CBH adsorption. As shown in Fig. 3a and b, *M*_*w*_ and PDI of lignin fractions were negatively correlated with binding strength between lignin and CBH (Pearson coefficient = − 0.911, *R*^2^ = 0.83, *p* value = 0.012 for *M*_*w*_; Pearson coefficient = − 0.922, *R*^2^ = 0.85, *p* value = 0.009 for PDI). Previous studies also pointed out that more uniform fragment size is favorable for the interaction of lignin with proteins [15]. Earlier studies on natural poplar variant showed that there was relationship between lignin *M*_*w*_ and glucose release [21], which was consistent with the present study.

It has been reported that hydroxyl groups, in particular phenolic hydroxyl groups, played an important role in cellulase binding to lignin [9]. Negative correlations of aliphatic hydroxyl group with cellulase adsorption [15] and hydrophobicity of lignin [12] were investigated in the previous studies. However, association between aliphatic hydroxyl group and CBH binding was not observed in this study. Instead, phenolic hydroxyl group in the lignin fractions showed a positive correlation with CBH adsorption ability. As shown in Fig. 3c, correlation between phenolic hydroxyl groups and binding strength was positive (*R*^2^ = 0.59, Pearson coefficient = 0.768, *p* value = 0.075), suggesting that content of phenolic hydroxyl group affected the binding strength between lignin from poplar and CBH. The results were consistent with previous studies [9, 10, 15]. It was reported that hydroxypropylation of phenolic OH can reduce the negative inhibitory effect of lignin on glucose release [9, 35]. Pretreated biomass with decreased phenolic OH was a contribution to an increased glucose yield [36]. Binding strength was negatively associated with enzymatic hydrolysis yield, indicating that greater binding strength between lignin and cellulase would result in less glucose yield [35]. It could be predicted that lignin fraction from natural poplar variant with more phenolic hydroxyl group would result in less glucose when cellulase hydrolysis was conducted. Li’s research with vanillin, which was from degradation of lignin, and three other compounds with similar structure showed that inhibitory effect on cellulase was from phenolic hydroxyl groups of vanillin [37]. Phenolic hydroxyl group could interact with cellulase by forming hydrogen bonding with amino acid residue and interfering cellulase hydrolysis to cellulose.

The influence of condensed aromatics on CBH adsorption was also observed in this study (Fig. 3d). A positive association between condensed aromatics and binding strength of lignin fractions to CBH (*R*^2^ = 0.62, Pearson coefficient = 0.787, *p* value = 0.063) indicated that lignin fractions with more condensed aromatics could bind stronger with CBH. Similarly, increase of cellulase adsorption with the degree of lignin condensation in the pretreated wood and bleached pulps was reported in the previous study [10]. In addition, Ko and coworkers reported that more condensed lignin coincided with increasing affinity of enzyme adsorption [16]. It is hypothesized that the increased degree of condensation would result in more cellulase adsorbed via hydrophobic interaction [38]. Furthermore, it was concluded that phenolic OH group in condensed syringyl and guaiacyl subunits have strong association with inhibitory effects on enzymatic hydrolysis [8]. However, only contents of condensed syringyl subunit showed a positive correlation between binding strength of lignin fractions and CBH (*R*^2^ = 0.54, Pearson coefficient = 0.735, *p* value = 0.096) in this study (Fig. 3e).

## Conclusions

The CBH adsorptions onto different lignin fractions from two *Populus trichocarpa* natural variants were investigated with the physicochemical properties of each lignin fractions. It was found that structural features of lignin have great effect on CBH binding. Also, molecular weight and PDI of lignin fractions were negatively correlated with CBH binding. The phenolic hydroxyl group content in the lignin fractions showed a positive correlation with CBH binding ability. In particular, the contents of total condensed aromatics and condensed syringyl of lignin fractions involved in the association of lignin with CBH likely do so by hydrogen bonding and hydrophobic interaction. The observation of correlations between lignin physicochemical properties and CBH adsorption in this study can be also used as a preliminary result for explaining the recalcitrance of *P. trichocarpa* natural variants.

## Methods

### Materials

Four-year-old *P. trichocarpa* natural variants were collected from a field site in Clatskanie, Oregon. The chemicals were purchased from Fisher (USA). Pronase was obtained from Sigma Chemical Company (USA). CBH was purchased by Megazyme (USA).

### Dilute acid pretreatment

*Populus trichocarpa* natural variants were debarked, Wiley-milled (screen size < 2 mm was used), and extracted by toluene/ethanol (2:1, v/v) for 8 h. The extractives-free material was then pretreated by a 1 l Parr pressure reactor (model 4560, Parr Instrument Company) with 0.5% sulfuric acid (v/v). The ratio of liquid to solid was 10:1. The pretreatment was kept at 160 ± 2 °C for 10 min (± 0.5 min). The stirring speed was set to 2.5 Hz. The heating rate was about 3 °C/min. The reactor was quenched in an ice water bath for 10 min to stop the pretreatment process. The pretreated solid residue was acquired by filtration and washed with an excess of deionized water until pH was neutral. The pretreated poplars were then air-dried overnight at room temperature.

### Fractionation of lignin from pretreated poplar natural variants

**Fig. 4:**
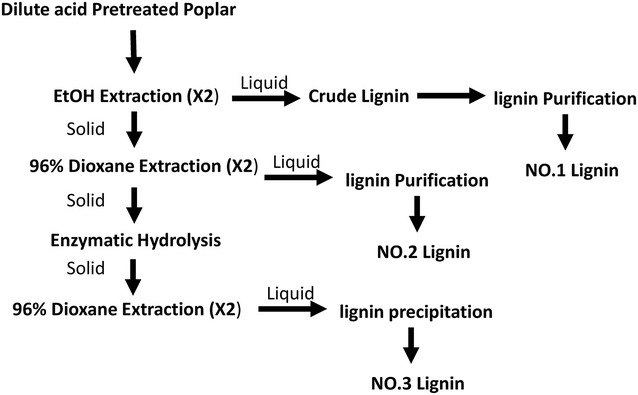
Fractionation of lignin from dilute acid pretreated poplar

Three different lignin fractions were separated from the dilute acid pretreated *P. trichocarpa* natural variants as presented in Fig. 4, following the method described before [39]. Pretreated *P. trichocarpa* natural variants were first extracted twice with ethanol for 24 h. The extract was rotary-evaporated and freeze-dried to obtain the crude NO.1 lignin (*L*_1_ and *H*_1_).

The solid residue after the ethanol extraction was air-dried and then extracted twice with 96% dioxane to provide crude NO.2 lignin (*H*_2_ and *L*_2_).

The air-dried residue after two-step extraction was hydrolyzed by overloading cellulase to remove carbohydrates. After protease treatment to remove any remaining protein, it was extracted by dioxane–water mixture (96:4, v/v; 10 ml/g biomass) to get the crude lignin *L*_3_ and *H*_3_. The crude lignin was purified according to the method of milled wood lignin (MWL) [40].

### Fourier transform infrared (FT-IR) analysis

FT-IR spectroscopy (Spectrum One FT-IR system, Perkin Elmer, Wellesley, MA) was employed to get the FT-IR spectra from 4000 to 650 cm^−1^. 64 scans with 2 cm^−1^ resolution were signal averaged and stored.

### Gel permeation chromatographic (GPC) analysis

Gel permeation chromatographic (GPC) analysis was conducted to determine the molecular weights of each lignin fraction. Lignin samples were acetylated prior to analysis according to method by Kumar [41]. Gel permeation chromatography was performed on an Agilent 1200 HPLC system (Agilent Technologies, Inc, Santa Clara, CA) with tetrahydrofuran (THF) as described in the previous study [42].

### Nuclear magnetic resonance (NMR) analysis

Around 50 mg lignin samples fraction were dissolved in 0.4 ml DMSO-*d*_6_. Bruker Avance III 400-MHz spectroscopy equipped with a 5-mm Broadband Observe probe (5-mm BBO 400 MHz W1 with Z-gradient probe, Bruker) was employed to conduct two-dimensional (2D) ^1^H–^13^C heteronuclear single quantum coherence (HSQC) NMR experiment at 298 K. A Bruker standard pulse sequence (‘hsqcetgpsi2’) was used with the following parameters: spectral width of 11 ppm in F2 (^1^H) with 2048 data points and 190 ppm in F1 (^13^C) with 256 data points; 96 scans and 1-s delay.

^31^P NMR spectra were obtained after derivatization of the lignin fractions with 2-chloro-4,4,5,5-tetramethyl-1,3,2-dioxaphospholane [28]. Endo *N*-hydroxy-5-norbene-2,3-dicarboxylic acid imide was used as the internal standard. The conditions for ^31^P NMR spectra were as follows: a 90° pulse angle, 25 s pulse delay, and 256 transients at room temperature.

### Adsorption of CBH onto lignin

CBH (Cel7A; Megazyme E-CBH I), supplied at 10 mg protein/ml, was from *Trichoderma longibrachiatum*. The binding of cellobiohydrolase I onto the lignin fractions was measured by the Langmuir isotherm protocol [43]. A range of concentrations of CBH were mixed with lignin samples (2%, w/v) and suspended in pH 4.8 acetic acid–sodium acetate buffer. The mixture was kept at 50 °C until the adsorption was constant. The PierceTM bicinchoninic acid (BCA) protein assay from Thermo scientific was used for determination of protein concentration. Adsorption parameters such as the maximum adsorption capacity (*E*_max_) and the equilibrium constant (*K*_ads_), were determined by linear regression of the adsorption data using the following equation.

$$\left[ {E_{\text{f}} } \right]/\left[ {E_{\text{ads}} } \right] = 1/\left( {K_{\text{ads}} \left[ {E_{ \text{max} } } \right]} \right) + \, \left[ {E_{\text{f}} } \right]/\left[ {E_{ \text{max} } } \right]$$

where [*E*_f_] (mg/ml) is the free protein concentration, [*E*_ads_] (mg/g) is the amount of protein adsorbed by the lignin, *K*_ads_ is Langmuir adsorption constant, and [*E*_max_] is the maximum amount of adsorbed protein.

## Additional file

**Additional file 1: Figure S1.** 2D-HSQC spectra and the main structures of the isolated lignins: (A) β-aryl-ether units (β-O-4); (B) phenylcoumarane; (C) resinols; (G) guaiacyl units; (S) syringyl units; (S’) oxidized syringyl units bearing a carbonyl at Cα; (PB) p-Hydroxybenzoate units. Condensed lignin was assigned from Sun et al. [8].

## Abbreviations

*H*: high recalcitrance poplar
*L*: low recalcitrance poplar
HSQC: heteronuclear single quantum coherence
CBH: cellobiohydrolase
FT-IR: Fourier transform infrared spectroscopy
S: syringyl
G: guaiacyl
GPC: gel permeation chromatography
*M*_*n*_: number average molecular weights
*M*_*w*_: weight average molecular weights
NMR: nuclear magnetic resonance
MWL: milled wood lignins
THF: tetrahydrofuran
HPLC: high performance liquid chromatography
DMSO-*d*_6_: deuterated dimethyl sulfoxide
TMDP: 2-chloro-4,4,5,5-tetramethyl-1,3,2-dioxaphospholane
PDI: polydispersity index
