# Supplementary material for: A structured understanding of cellobiohydrolase I binding to poplar lignin fractions after dilute acid pretreatment
Source: Biotechnol Biofuels. 2018 Apr 4;11:96. doi: 10.1186/s13068-018-1087-y (PMC5883885; doi:10.1186/s13068-018-1087-y)
Supplement: Supplementary file 1 — Additional file 1: Figure S1. 2D-HSQC spectra and the main structures of the isolated lignins: (A) β-aryl-ether units (β-O-4); (B) phenylcoumarane; (C) resinols; (G) guaiacyl units; (S) syringyl units; (S’) oxidized syringyl units bearing a carbonyl at Cα; (PB) p-Hydroxybenzoate units. Condensed lignin was assigned from Sun et al. [8]. [file 13068_2018_1087_MOESM1_ESM.docx]

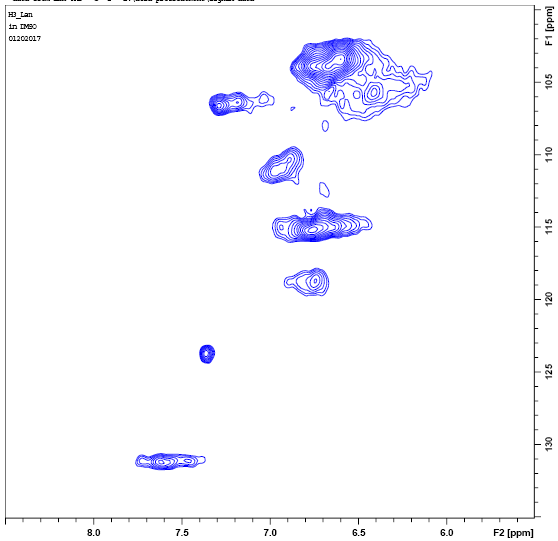

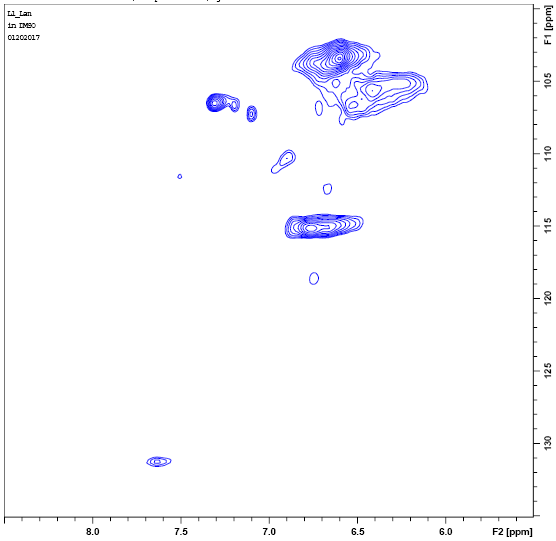

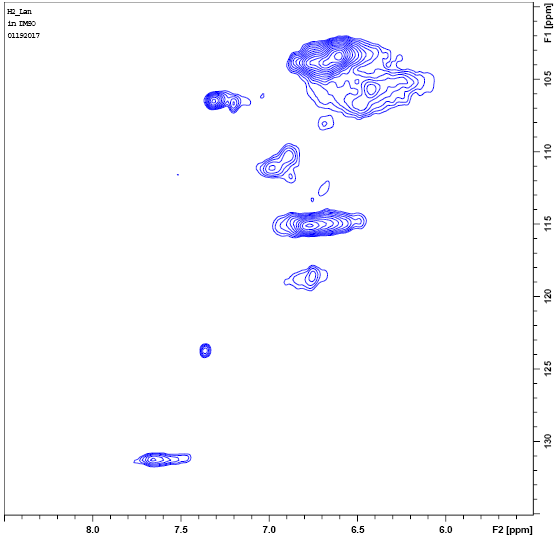

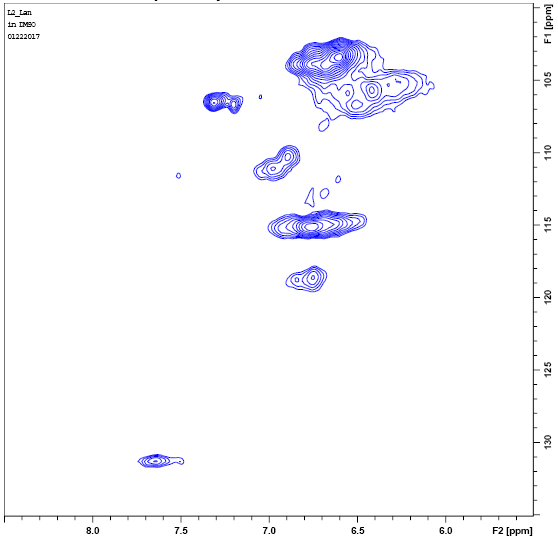

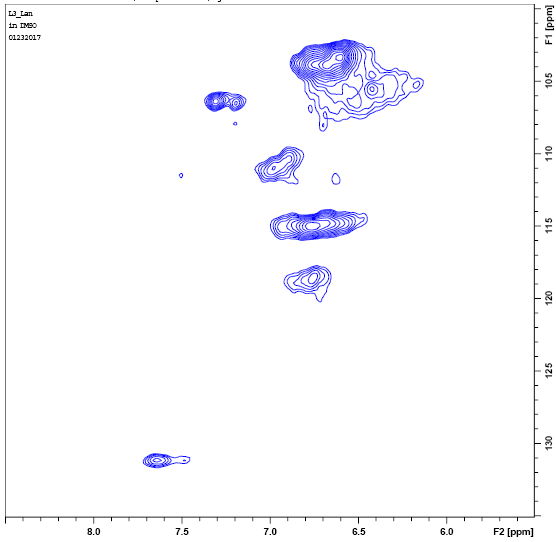

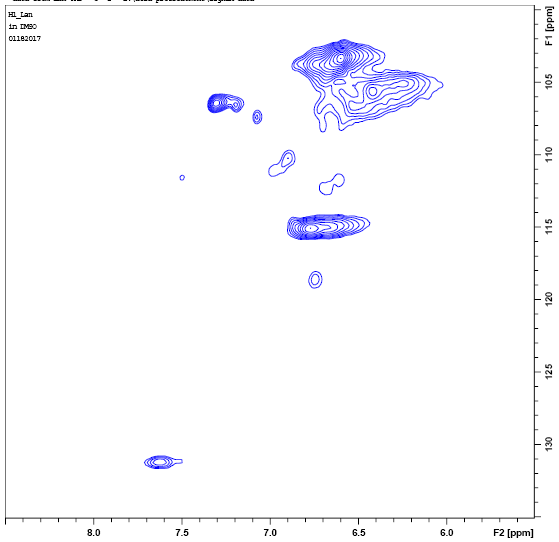


Condensed

lignin

Condensed

lignin

PB2/6

G5

G6

G2

S’2/6

S2/6

H3 lignin

PB2/6

G6

G5

G2

S’2/6

S2/6

H2 lignin

H1 lignin

PB2/6

G5

G6

G2

S’2/6

Condensed

lignin

PB2/6

G5

G6

G2

S2/6

S’2/6

L3 lignin

L2 lignin

S’2/6

G2

G5

G6

PB2/6

Condensed

lignin

PB2/6

G6

G5

G2

L1 lignin

S2/6

S’2/6

S2/6

S2/6

Condensed

lignin

S’2/6

Condensed

lignin


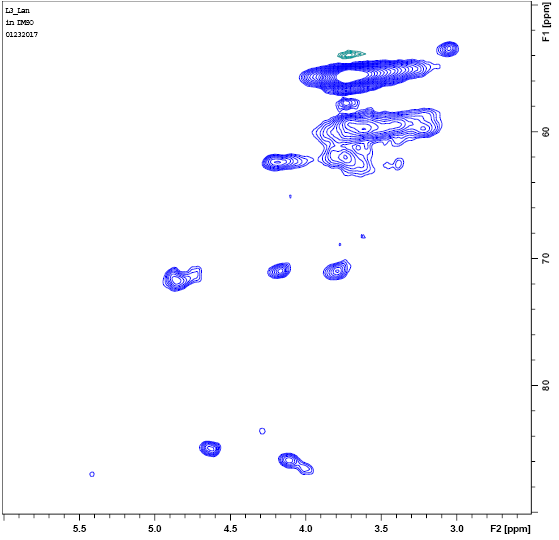

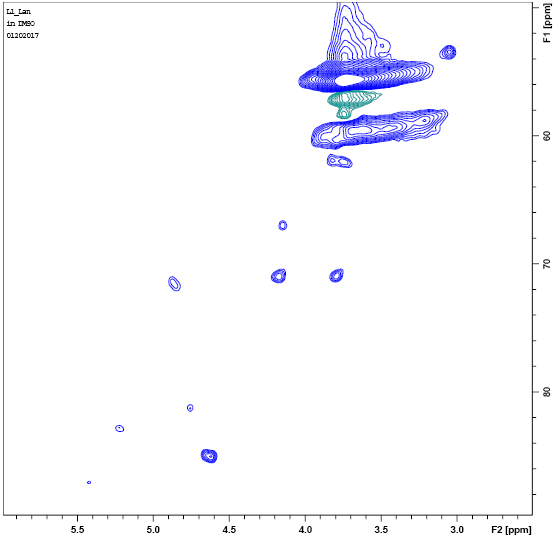

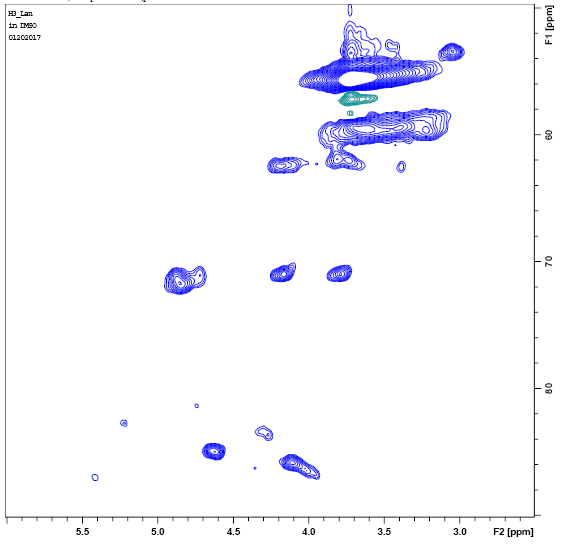

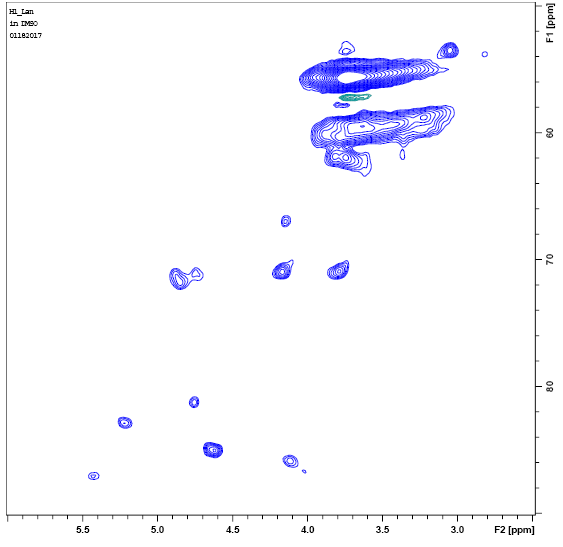

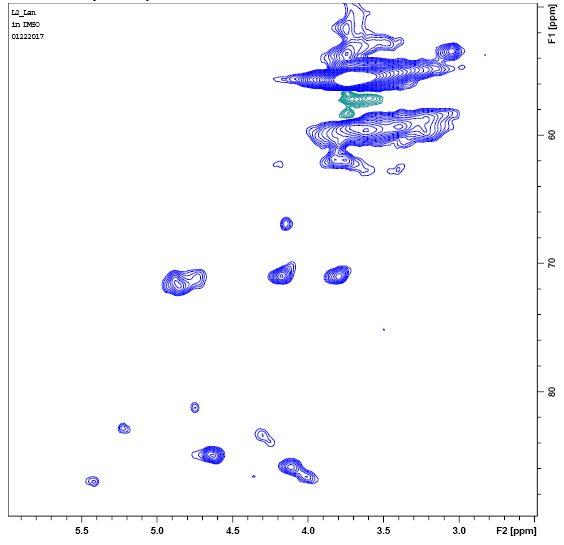

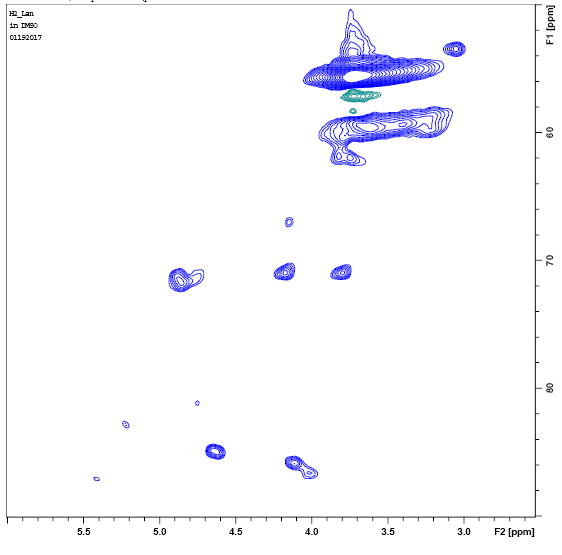


A_β_

Cα

Bα

Cγ

Aα

Bγ

Aγ

C_β_

Methoxyl

H3 lignin

C_β_

Methoxyl

Aγ

Bγ

Cγ

Aα

A’β

Cα

A_β_

Bα

H2 lignin

C_β_

Methoxyl

Aγ

Bγ

Cγ

Aα

A’β

Cα

Bα

H1 lignin

C_β_

Methoxyl

L3 lignin

Bγ

Aγ

Cγ

Aα

A’β

A’β

A_β_

Cα

Bα

Aγ

Methoxyl

Bγ

Cα

Cγ

Aα

A_β_

Cα

Bα

C_β_

L2 lignin

Bγ

Bα

A_β_

A’β

Cγ

Aα

C_β_

Aγ

Methoxyl

L1 lignin

Figure S1. 2D-HSQC spectra and the main structures of the isolated lignins: (A) β-aryl-ether units (β-O-4); (B) phenylcoumarane; (C) resinols; (G) guaiacyl units; (S) syringyl units; (S’) oxidized syringyl units bearing a carbonyl at Cα; (PB) p-Hydroxybenzoate units. Condensed lignin was assigned from Sun S, Huang Y, Sun R, Tu M: Strong association of condensed phenolic moieties in isolated lignins with their inhibition of enzymatic hydrolysis. Green Chem 2016, 18, 4276–4286.
